# Genomics of Maize Resistance to Fusarium Ear Rot and Fumonisin Contamination

**DOI:** 10.3390/toxins12070431

**Published:** 2020-06-30

**Authors:** Rogelio Santiago, Ana Cao, Rosa Ana Malvar, Ana Butrón

**Affiliations:** 1 Departamento de Biología Vegetal y Ciencias del Suelo, Facultad de Biología, Universidad de Vigo, As Lagoas Marcosende, Agrobiología Ambiental, Calidad de Suelos y Plantas (UVIGO), Unidad Asociada a la MBG (CSIC), 36310 Vigo, Spain; rsantiago@uvigo.es; 2 Misión Biológica de Galicia (CSIC), Apdo. 28, 36080 Pontevedra, Spain; anacao@mbg.csic.es (A.C.); rmalvar@mbg.csic.es (R.A.M.)

**Keywords:** Fumonisin, Fusarium ear rot (FER), *Fusarium verticillioides*, maize, host resistance genomics

## Abstract

Food contamination with mycotoxins is a worldwide concern, because these toxins produced by several fungal species have detrimental effects on animal and/or human health. In maize, fumonisins are among the toxins with the highest threatening potential because they are mainly produced by *Fusarium verticillioides*, which is distributed worldwide. Plant breeding has emerged as an effective and environmentally safe method to reduce fumonisin levels in maize kernels, but although phenotypic selection has proved effective for improving resistance to fumonisin contamination, further resources should be mobilized to meet farmers’ needs. Selection based on molecular markers linked to quantitative trait loci (QTL) for resistance to fumonisin contamination or/and genotype values obtained using prediction models with markers distributed across the whole genome could speed up breeding progress. Therefore, in the current paper, previously identified genomic regions, genes, and/or pathways implicated in resistance to fumonisin accumulation will be reviewed. Studies done until now have provide many markers to be used by breeders, but to get further insight on plant mechanisms to defend against fungal infection and to limit fumonisin contamination, the genes behind those QTLs should be identified.

## 1. Introduction

Food and feed contamination with mycotoxins is a worldwide threat for animals and humans, especially in warm and humid regions [1]. Mycotoxins are low molecular weight secondary metabolites derived mainly from amino acids, shikimic acid, or malonyl coenzyme A that elicit toxic responses in higher vertebrates and/or humans when ingested at low concentrations. They are produced by certain molds and can accumulate on a variety of different crops and foodstuffs such as cereals, nuts, spices, raisins, dried fruits, apples, and coffee beans [2]. Over 300 mycotoxins have been identified, mainly produced by fungus species of the genera *Aspergillus*, *Fusarium*, and *Penicillium*, in which each particular mycotoxin is produced by several fungal species or being specific to a single species. Maize is among the most commonly contaminated commodities [3]. Although several mycotoxins can contaminate maize kernels, fumonisins mainly produced by *F. verticillioides* and *F. proliferatum* are the most common mycotoxins due to the wide distribution of *F. verticillioides* from the tropics to warm temperate regions [4,5,6]. A detailed and updated overview of research advances on *F. verticillioides* biology, maize–*F. verticillioides* interaction and the molecular underpinnings of pathogenicity and environmental fitness can be found in the referred review [7].

The consumption of fumonisin contaminated food and feed can cause important disorders in humans and animals, respectively. The main health disorders in animals are leukoencephalomalacia in horses, pulmonary edema in swine, liver and heart damage in both, hepatic necrosis, and in the long run, kidney and liver cancer in rodents, and growth and liver disorders in poultry and cattle, respectively [8,9,10]. The effects of fumonisins on human health are not clearly established, although epidemiological studies have suggested a likely relationship between the intake of fumonisin-contaminated maize and incidence of esophageal cancer and occurrence of neural tube defects in human embryos [11,12,13,14,15]. Fumonisins have been classified as possibly carcinogenic to humans by the International Agency for Research on Cancer [16]. Updated information on fumonisin discovery history, chemical definition, and toxic effects can be found in a recently published book chapter [5]. As exposure to fumonisins needs to be kept as low as possible, many countries have regulations that establish maximum levels permitted or acceptable for different feed and foodstuffs. The Food and Drug Administration (FDA) of the USA delivered a document in 2001 with recommended values for fumonisin content [17], but some other countries such as South Corea or Indonesia have issued regulation on allowed maximum values [18] as well as international organizations such as the European Union [19].

Kernel infection by *F. verticillioides* and fumonisin accumulation occurs in the field, and those events are greatly affected by environmental conditions. However, significant differences in kernel infection and fumonisin accumulation have been found among maize genotypes, and resistance-related traits are moderately heritable [20,21,22,23,24,25,26,27,28,29,30,31,32,33,34,35,36,37]. Consequently, plant breeding has emerged as an effective and environmentally safe method to reduce fumonisin levels [38,39]. Moreover, as fumonisin quantification is expensive and labor consuming, the visual estimate of kernel infection known as Fusarium ear rot (FER) score has emerged as a suitable trait for performing indirect selection based on the reported high genotypic correlation coefficients between FER and fumonisin content across different environments and genetic backgrounds [30,33,40,41].

The breeding programs implemented to increase resistance to FER have taken into account additive effects because those effects have been reported as the most important for the inheritance of resistance to FER and fumonisin accumulation [42,43,44,45,46]. However, it is should be pointed out that dominant as well as epistatic effects could also be involved in the inheritance of these traits. Backcross breeding was used to transfer resistance to FER from an unadapted donor inbred (GE440) to a susceptible commercial inbred (FR1064), and the strategy was effective for improving quantitative disease resistance of the released maize lines but gains in topcross performance were not achieved [47]. Similarly, a pedigree selection approach to breed inbred lines with increased resistance to FER and fumonisin accumulation resulted in maize genotypes more resistant to fumonisin accumulation in some environments, but not in all [48]. Recurrent selection has also proven its efficacy, since three cycles of recurrent selection for reduced FER and increased yield in a synthetic population (ReFus) partially resistant to FER attained significant gains for resistance to FER and indirectly for resistance to fumonisin accumulation in the population per se and testcrossed [49]. However, as Chiuraise et al. [50] pointed out, although high genetic gains for resistance to fumonisin contamination could be realized by phenotypic selection, further resources should be mobilized to upscale the effort to breed new varieties to cope with farmers’ requirements. 

**Figure 1:**
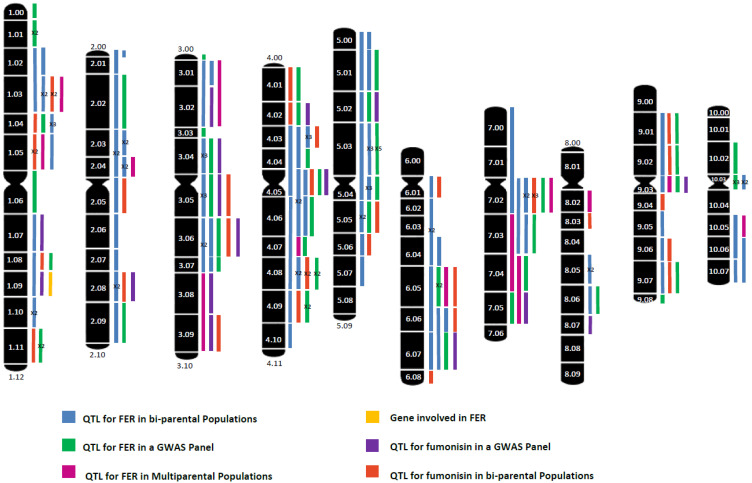
Bin localization of quantitative trait loci (QTL) and genes reported in the bibliography as involved in resistance to Fusarium ear rot (FER) and fumonisin contamination. QTL co-localizations in different populations using the same mapping approach are marked by ×2, ×3 and ×4 corresponding to co-localizations in two, three, and four populations, respectively.

Selection based on the phenotype could be effective, but it faces several problems other than being labor and time-consuming. Phenotyping maize for FER resistance requires (1) field trials conducted in several environments due to the large genotype and environment interaction, and (2) the proper management of inoculation timing due to variation among genotypes for flowering [51]. Those difficulties, in addition to nowadays increasing availability of dense and cheap molecular marker sets, has prompted to move the focus from conventional to marker-assisted and genomic selections, although results from breeding programs for increased maize resistance to FER implementing molecular tools are not yet published [51,52]. In this paper review, most studies oriented to identify genomic and/or genes and pathways implicated in resistance to FER and fumonisin accumulation are revised. Studies done until now have provide many markers to be used by breeders, but to get further insight on plant mechanisms to defend against fungal infection and to limit fumonisin contamination, the genes behind those QTLs should be validated. A graphical summary of bin localizations of QTL reported in the literature (Table 1) for resistance to FER and fumonisin contamination is shown in Figure 1.

## 2. Mapping of QTL for FER and/or Fumonisin Content in Bi-Parental Populations

The pioneering work by the International Maize and Wheat Improvement Center’s (CIMMYT) researchers showed that in two F_2:3_ mapping populations derived from crosses between white inbred lines adapted to Mexican highlands, QTL for FER could be found in chromosomes 1, 2, 3, 4, 5, 6, 7 and 10 [43]. Those QTL could explain from the 11% to 44% of the phenotypic variance for FER; 13 displayed significant QTL × environment interactions, and only three overlapped in both populations. Based on those results, authors warned about using marker-assisted selection for improving FER resistance. 

Similar conclusions were drawn by Robertson-Hoyt [53], as they found many minor QTL for FER and fumonisin content with low consistency across populations, but in general, these were stable across environments. However, as fumonisin determinations are labor-demanding and expensive, the same authors recommend marker-assisted backcrossing. More recent studies reported that although most QTL for FER are minor QTL, some major QTL whose effects on FER were validated by near-isogenic lines could be managed by marker-assisted breeding [54,55,56,57]. In addition, as the number of mapping populations explored increased, the overlapping of QTL for FER found in different populations started to be more common [55,58,59]. 

Maschietto et al. [60] found 15 QTL for FER and 17 QTL for fumonisin contamination; eight of them were common to both traits and five were co-located with QTL for ear rot caused by other fungi. These results confirmed the high genetic correlation between FER and fumonisin content previously reported and the existence of genomic regions containing QTL for multiple ear rot resistances that are stable across environments [59,61]. In a recently published QTL study, different recombinant inbred line (RIL) families from a nested association mapping (NAM) population have been used to elucidate the genetic mechanisms underlying ear-mediated resistance to FER and fumonisin accumulation as well as to discover QTL for FER and fumonisin content [58]. These authors identified 23 novel QTL for resistance to infection by *F. verticillioides* and to fumonisin accumulation and suggested that seven of them could be mediated by kernel and cob architecture. They also confirmed that the trait kernel density was a good proxy of fumonisin content and suggested that larger and less dense cobs could contribute to reduced FER scores and fumonisin levels [62]. Wu et al. [63] re-evaluated the RIL population initially evaluated by Li et al. [56] and detected 10 QTL for FER; QTL located in bins 4.05-4.08 were validated using NILs. 

As summary of this section, in the last 15 years, a limited number of bi-parental mapping populations have been used to explore the genetic architecture of maize resistance to FER and even fewer bi-parental populations to dissect genomics of resistance to kernel contamination with fumonisins. Bi-parental mapping populations are still useful tools for genetic studies, but the rapid development of high-throughput genotyping technologies permit now efficiently using other mapping populations that offer higher QTL resolution and explore wider genetic variability. 

## 3. Genome-Wide Association Studies for FER and Fumonisin Content

Most genome-wide association studies (GWAS) have been focused rather on genomics related to FER than on genomics involved in resistance to fumonisin content, as we next review. The first reported GWAS for FER used a panel of 267 inbreds and approximately 50,000 single nucleotide polymorphisms (SNPs) and detected few QTL, each one with small effects on phenotypic variation for FER [64]. Inbreds belonging to tropical and popcorn subgroups tended to perform better than inbreds from other groups, and the authors proposed some genes related to programmed-cell death as candidate genes for those QTL. As results suggested that many genes with small effects would underline the inheritance of FER and linkage disequilibrium (measured as r^2^) drops below 0.1 beyond 10 kpb, authors pointed out that the low density of markers and the limited number of inbred studied could hamper QTL detection. In a more recent paper, a panel of 1687 inbreds genotyped for around 200,000 SNPs was used for performing GWAS for FER [65]. Nine significant SNPs were located corresponding to seven QTL, alleles conferring resistance at significant SNPs being rare and more frequent in tropical than in temperate inbreds. The same authors also performed GWAS with a panel of 556 hybrids resulting from crossing some inbreds to one or two testers, but no significant SNP for FER was detected, because genetic variability for FER in hybrids is reduced compared to inbreds. Both studies reported substantial additive polygenic variation for ear rot resistance, suggesting that phenotypic and genomic selection approaches should be useful for improving resistance to FER. However, breeding effectiveness will depend on performing accurate phenotypic evaluations of resistance either for phenotype direct selection or for building reliable genomic selection models. 

Therefore, as resistance variants were more frequent among tropical inbreds, it is not casual that subsequent GWAS were done using tropical inbreds. Chen et al. [57] performed GWAS using an inbred panel of 940 tropical inbreds, 63 of which were highly resistant to FER. These authors found 45 SNPs significantly associated with FER at *p* < 10^−3^ and 15 haplotypes; they reported small effects for each SNP or haplotype and wide co-localization with QTL reported in previous studies. Among the candidate genes proposed, they highlighted a gene encoding a glucose/ribitol dehydrogenase because it belongs to a subset of short-chain dehydrogenases and reductases involved in pathogen toxin reduction.

In a later work, Coan et al. [66] also explored genomics associated to FER in a panel of 183 tropical inbreds; 14 SNPs out of the available 268,000 SNPs were significantly associated with FER. Each SNP explained from 15% to 25% of phenotypic variance in individual environments, but there were no consistent SNP–trait associations across the three environments. Four genes associated to significant SNPs encode defense-related proteins, including a gibberellin 2-oxidase4, a glucosyltransferase, a Ras-related protein RHN1, and an anthranilate phosphoribosyltransferase. Similarly, de Jong et al. [67] showed that significant DarT-seq markers explained 37–51% of the phenotypic variability in each environment, but few coincidences between markers significantly associated with the percentage of ears with rot symptoms in joint and individual analyses, pointing out large QTL × environment interaction effects. These authors also reported few co-localizations with QTL reported in previous studies but that could be consequences of the different inbred panels, marker distribution, and disease-related traits used. de Jong et al. [67] performed GWAS analyses with the percentage of ears per plot presenting rot symptoms instead of using a visual score to assess ear damage extension in a representative sample of ears, as it has been commonly done in most studies. The percentage of ears presenting symptoms is not appropriate to accurately estimate damage spread, because no differentiation is made between ears with few kernels presenting rot symptoms and ears totally rotten. These authors suggest some candidate genes for the associated markers to the percentage of damaged ears, such as a gene responsible for the innate immune response that belongs to the class of resistant-genes that contain nucleotide binding site and leucine rich repeat (NBS-LRR) receptors, two transcription factors (*nactf11* and *nactf61*), and genes responsible for the oxidation-reduction process and peroxidase activity. The most recent GWAS for FER has been implemented using a 265 inbred panel and has located 18 SNP significantly associated with FER [63].

A recently published GWAS on resistance to FER was performed using a multi-parent advanced generation intercross (MAGIC) population resulting in 13 putative minor QTL for FER [68]. The MAGIC population was composed of RIL derived from crosses among eight temperate inbreds with different levels of resistance to FER. This population could be a good complement to diverse inbred panels in the quest to uncover genomic regions implicated in FER resistance among temperate materials because frequencies of favorable alleles for resistance to FER are low in inbred panels, especially in temperate inbred groups. The authors showed that among the uncovered QTL, the QTL in bins 7.04–7.05, 8.02, and 9.03 were highly reliant. Novel QTL were found but most QTL overlapped with QTL for FER and fumonisin contamination previously located. 

So far, there is just one GWAS on resistance to fumonisin accumulation, which has confirmed that highly significant genotype × environment interaction as well as genetic variation for many genes with small effects would contribute to the high complexity of the inheritance of maize resistance to kernel contamination [69]. In the inbred panel of approximately 250 entries, 39 SNPs, clustered into 17 QTL, were significantly associated with resistance to fumonisin accumulation in maize kernels. The high resolution of QTL identified allows proposing candidate genes for those QTL, many candidates being implicated in maize immune response signaling. 

In brief, the genomic studies on FER and fumonisin contamination show that the value of selection on identified markers for improving these characters is limited because most genetic variability has not been captured by QTL models due to the highly polygenic nature of resistance to FER and to fumonisin contamination. Therefore, a general recommendation can be made in which selection for particular resistance alleles could be useful as long as genomic selection for polygenic background, either for target or adaptation traits, is also performed. In addition, adaptation should be also an important issue, because resistance alleles are more frequently found in tropical maize varieties, which are not adapted to temperate regions. 

The infection of maize kernel by *F. verticillioides* can occur via several routes: through silks, pericarp wounds, or via systemic infection from the seed [70,71,72]. However, most kernel infections are due to the entrance of the fungus through silks and/or pericarp wounds. In systemic infection, the seed-transmitted fungus develops inside the young plant, after which it moves from the roots to the stalk and finally to the cob and kernels [73]. The resistance of maize to seed infection at germination and seedling (FSR) has been also studied using GWAS approximations, but no genetic correlation was found between FER and FSR [70,74,75,76]. These results suggest that mechanisms involved in both types of infections would be different, and moreover, susceptibility at the seedling stage would not have an important impact on FER. Some studies have shown that the cob could play an important role in the spread of kernel rot because local infection through silks could progress directly to kernels and/or via the cob [70,77]. Therefore, resistant cobs can block the fungi from entering the kernels and reduce the incidence of diseased kernels. However, resistance to FER is not correlated with resistance to cob rot under kernel artificial inoculation conditions or natural conditions that favor kernel direct infection through wounds. Therefore, cob and kernel resistance factors to infection by *F. verticillioides* would complement each other to reduce FER. Mu et al. (2019) [77] used several approximations to dissect genomics involved in Fusarium cob resistance: GWAS in a panel of 258 inbreds for high resolution mapping; the validation of QTL using BC4F1 near-isogenic lines, and expression profiles of candidate genes in resistant and susceptible inbreds. 

Although GWAS have contributed to find high-resolution QTL and many candidate genes have been proposed for those QTL, so far, only one gene implicated in resistance to FER have been cloned: the gene *ZmAuxRP1*, which encodes a plastid stroma-localized auxin-regulated protein [78]. This gene was confirmed as the causal gene of a QTL for resistance to stalk rot by *F. graminearum* and has shown a quick response to pathogen challenge with a rapid yet transient diminution in expression that led to arrested root growth but enhanced resistance to Gibberella stalk rot and FER. The authors suggested that *ZmAuxRP1* may be involved in the regulation of resource allocation toward growth or immune response by diverting substrates to Indole-3-acetic acid (IAA) or benzoxazinoid biosynthesis, respectively. However, this hypothesis has to be verified because *F. verticillioides* is one of the Fusarium species that has the ability of benzoxazinoid detoxification [7,79].

## 4. Gene Expression Studies 

Besides GWAS approaches using DNA markers, other molecular tools can be used to study genomics involved in resistance to FER and to contamination with fumonisins. Gene expression approaches has been extensively used to dissect genes involved in maize response to kernel infection by *F. verticillioides*. Several studies compared the response of resistant and susceptible maize inbreds to infection; these studies differ in the molecular technique [from microarrays to RNA-seq], genotypes, and post-inoculation times [from 12 to 120 h] used [80].

Lanubile et al. [81] studied the differential gene expression in silks and kernels between infected and non-infected field plants of lines with contrasting levels of resistance to FER [CO354 as susceptible and CO441 as partially resistant]. The authors highlighted that similar functional categories of genes were involved in the response to infection of both inbreds, although, in the resistant inbred, the defense-related genes assayed were transcribed at high levels before infection and provided basic defense to the fungus, while expression changes in inoculated kernels were stronger in the susceptible genotype. They suggested that responses upon *F. verticillioides* infection involve changes in the expression of a large number of maize genes, including genes implicated in reprogramming cellular metabolism, the accumulation of barrier-forming substances (reinforcement of cell walls), and the production of antimicrobial compounds that act directly to prevent pathogen invasion. In a later study, Lanubile et al. [82] corroborated, under controlled greenhouse conditions, that basal defense could be a principal contributor to resistance. However, in another study conducted by the same research group [83], it was found that upon point infection, the resistant line was more efficient at activating a systemic response. These authors indicated that besides basal defenses, earlier systemic activation of defense and regulatory genes, such as chitinases, glucanases, pathogenesis-related (PR) proteins, and positive and negative transcriptional factors such as MYB-like and WRKY1 could contribute to reduce fungal growth in the resistant genotype. 

Chinese researchers working with two different inbreds with contrasting resistance to *F. verticillioides* (Bt-1, resistant, and Ye478, susceptible) suggested that the constitutively elevated expression of defense genes in maize husks could play important roles in modulating the response to infection by *F. verticillioides* enhancing the plant protection system; in that particular study, more genes were up-regulated by *F. verticillioides* infection in the resistant than in the susceptible inbred [84]. In a later study, the same research team showed stronger activity response of defense enzymes in the susceptible than in the resistant inbred and highlighted some genes as possible regulators of the differential response [85]. In another study, no important changes in transcriptional and metabolomic profiles were detected in the silks and kernels of the resistant line (L4637) compared to the susceptible inbred (L4674) following *F. verticillioides* inoculation, which supports the hypothesis that constitutive defense mechanisms may confer partial resistance against *F. verticillioides* infection [86].

Microarray approaches were replaced by next-generation RNA-sequencing approaches in subsequent expression studies. Lanubile et al. [87] re-examined the responses of inbreds CO441 and CO354 to *F. verticillioides* infection and found differences in basal gene expression between both inbreds, CO441 kernels showing higher levels of expression of genes distributed over all functional classes. A similar response to inoculation was observed in both genotypes, although the magnitude of induction was much greater in the resistant genotype. This response included the higher activation of genes involved in pathogen perception, signaling, and defense, including WRKY transcription factors and jasmonate/ethylene-mediated defense responses, and genes related to shikimate, lignin, flavonoid, and terpenoid pathways. 

Later on, Wang et al. [88] used RNA-seq to study the response of the resistant inbred BT-1 to *F. verticillioides* infection and found that multiple genes involved in abscisic acid (ABA), salicylic acid (SA), and jasmonic acid (JA) hormone signaling pathways were induced as well as genes involved in pathogen-associated molecular pattern-triggered immunity (PTI) but not effector-triggered immunity genes. These results suggest that PTI could play a major role in the resistance of BT-1 to *F. verticillioides*, and several hormone signaling would be involved. Furthermore, the authors pointed out that many differentially expressed genes (DEG) between control and inoculated kernels could be mapped to known QTL for FER, agreeing with previous results using microarray experiments [89]. The DEG in these known QTL could be considered as valuable candidate genes and should be further studied to determine their role in resistance. Following this idea, Maschietto et al. [60] used the previous RNA-seq characterization of CO441 and CO354 after inoculation with *F. verticillioides* and at control conditions [87], and the DEG between both inbreds that mapped within the confidence interval of QTL for FER and/or fumonisin content in a mapping population derived from the cross CO441 × CO354 were proposed as candidate genes for those QTL. The proposed genes codify for thioredoxin (YPTM1), lypoxygenase (LOX8), heat shock proteins, transcription factors (WRKY74 and AP2/ERF), S-adenosylmethionine decarboxylase, carbohydrate transmembrane transporter, vicilin-like antimicrobial peptide 2–3 (AMP2-3), ascorbate peroxidases, and glutathione transferases.

Simultaneously to genome-wide expression studies, research focused on the expression changes of particular defense genes also provided useful knowledge about genes or pathways that could be involved in resistance to FER and to fumonisin contamination. Blacutt et al. [7] suggested that maize defense against *F. verticillioidesis* follows the typical necrotrophic fungus interaction pattern, in which the plant exhibits nonspecific responses such as the induction of pathogenesis-related (PR) proteins [90,91]. Consequently, Maschietto et al. [92] investigated the changes in kernel RNA expression due to *F. verticillioides* infection in two resistant (CO441 and CO433) and two susceptible (CO354 and CO389) maize inbreds for pathogenesis-related (*PR1, PR5, PRm3, PRm6*) and reactive oxygen species (ROS) scavenging (peroxidase, catalase, superoxide dismutase, and ascorbate peroxidase) genes. In addition, the oxidation level and the enzymatic activity of ascorbate–glutathione cycle, catalase, superoxide dismutase, and cytosolic and wall peroxidases were explored. The control kernels of the resistant lines showed higher gene expression and enzymatic activities, highlighting the key role of constitutive resistance in limiting pathogen progression. In contrast, defensive genes were more highly induced by pathogen inoculation in the susceptible inbreds, but these inbreds still showed less antioxidant activity than resistant ones, resulting in increased levels of H_2_O_2_ and lipid peroxidation. Similar results were also reported in a study under inoculation with *F. subglutinans, F. proliferatum,* and *A. flavus* confirming the overlapping of genes or pathways contributing to resistance to different mycotoxin-producer pathogens [93]. 

In addition, to set the focus on genes directly involved in defense, the study of genes involved in maize response signaling has deserved special attention. Cross-kingdom signaling molecules appear to play a significant role in determining the outcomes of the *F. verticillioides* interaction with maize; therefore, the understanding of the molecular signaling occurring during maize–*F. verticillioides* interactions could pave the way for the discovery of genes involved in resistance or susceptibility, and several studies have targeted maize genes involved in response signaling to *F. verticillioides* infection and/or on fungal genes implicated in the interaction [7,94]. Real-time RT-PCR experiments for 15 maize lipoxygenase (*LOX*) genes were performed on kernels of inbreds CO441 and CO354 collected at 3, 7, and 14 days post-inoculation (dpi) with the fungus [95]. *LOX* genes were stronger and earlier induced after inoculation by *F. verticillioides* in the resistant inbred, corresponding with the mounting of resistance between 7 and 14 dpi that limited fungal growth and fumonisin accumulation in that inbred compared to the susceptible one. Therefore, authors concluded that the resistant inbred could activate more efficiently the defense response that would depend on an overexpression of LOX pathway genes and suggested a key role for JA in resistance to *F. verticillioides*. The impact of maize oxylipins on mycotoxin production would be mediated through changes in the transcription of fumonisin biosynthetic genes [96]

Later on, Fauguel et al. [97] suggested that *F. verticillioides* could use compounds from the maize 9-LOX pathway to promote infection in kernels while maize volatiles produced by the 13-LOX pathway could be associated to maize field resistance. They firstly studied, in *in vitro* experiments, the effects of volatile organic compounds (VOCs) of silks and kernels from genotypes with different levels of resistance to FER and found that VOCs from the most resistant and susceptible genotypes inhibited and promoted, respectively, fungal growth. Then, kernel and silk VOC profiles of different maize genotypes were studied; the most susceptible genotypes produced large amounts of VOCs with a prevalence of C9 compounds, indicating that these volatiles might be associated with the fungal growth promotion observed. In addition, real-time PCR for several *lipoxygenase* transcripts in silks of two inbreds with contrasting levels of resistance revealed that *13-LOX* gene expression seems to be higher in the moderately resistant inbred while *9-LOX* gene expression, which was stimulated by inoculation, was higher in the susceptible inbred. These results agreed with previous findings by Gao et al. [98] that showed how the disruption of a *9-LOX* gene, *ZmLOX3*, resulted in reduced levels of several 9-LOX-derived hydroperoxides and reduced conidiation and production of fumonisin B1 by *F. verticillioides*. However, Christensen et al. [99] demonstrated that a unique monocot-specific 9-LOX plays a key role in defense against *F. verticillioides* in diverse maize tissues. They also provided genetic evidence about the involvement of the hormone JA in maize defense against this pathogen, because the mutator transposon-insertional *lox12-1* mutant showed reduced resistance to infection by *F. verticillioides* and higher fumonisin accumulation accompanied by diminished levels of the JA precursor 12-oxo phytodienoic acid, JA-isoleucine, and the expression of jasmonate-biosynthetic genes [99]. Moreover, Battilani et al. [94] have recently revealed that *9-LOX* genes could be implicated in susceptibility as well as in defense. *ZmLOX3* was confirmed as a major susceptibility factor induced by fungal oxylipins, because *ZmLOX3*-mediated signaling promotes the biosynthesis of fungal virulence-promoting oxylipins, but *ZmLOX3* suppresses JA-stimulating 9-LOXs such as *ZmLOX4*, *ZmLOX5*, and *ZmLOX12*, which are essential for JA-mediated defense. These results would agree with previous expectations made by Scala et al. [100]. These authors suggested that maize reacts by producing oxylipins to interfere with pathogen invasion in which *F. verticillioides* oxylipins try to reprogram pathogen-triggered immunity. However, in some cases, some maize oxylipins favor pathogen virulence, inducing effector-triggered susceptibility.

As the down-regulation of *ZmLOX3*, a known susceptibility factor, was recently reported as a mechanism behind *Trichoderma virens*-mediated induced systemic resistance [101], studies of induced systemic resistance (ISR) activated by beneficial fungus could be also a valuable resource to expose hidden molecular mechanisms essential to reduce disease development. In that sense, the study of ISR activated by the beneficial fungus *T. atroviride* in maize plants that were posteriorly inoculated with *F. verticillioides* have provided evidence of the role of pathogenesis-related, plant cell-wall reinforcement, fungal cell-wall-degrading enzymes, and secondary metabolism in maize immune response to *F. verticillioides* [102].

## 5. Reverse Genetics to Uncover Metabolic Pathways Involved in Resistance to *F. verticillioides* and Fumonisin Contamination

Another procedure to unravel metabolic pathways involved in resistance to *F. verticillioides* and fumonisin contamination would be to check the effect of pathway disturbance on resistance. Disrupting mutants and isogenic wild-type counterparts would be compared to assess the influence of the particular pathway on resistance.

Secondary metabolites with antioxidant properties, mainly terpenoids and phenylpropanoids have frequently been reported as probably involved in plant defense against fungal pathogens, and in addition to antifungal properties, they could interfere in mycotoxin biosynthesis [103]. Therefore, it is not surprising that known mutants for genes implicated in terpenoid and phenylpropanoid pathways has been assayed along with their near-isogenic wild-type counterparts to validate if they have any significant effect on resistance to FER and/or fumonisin contamination.

Venturini et al. [104] studied the performance of two isogenic hybrids, one having pigmentation in the pericarp (conferred by allele *P1-rr* at the *P1* gene) and the other without (allele *P1-wr* that disrupts the flavonoid pathway). The pigmented hybrid showed lower values for FER symptoms and fumonisin contamination, although the difference was not always significant, suggesting that the contribution of flavonoids to fumonisin resistance would depend on environmental conditions such as, for example, the incidence of kernel damage by insects.

Using an array of commercial maize hybrids, Christensen et al. [105] found a negative correlation between total terpenoids and fungal growth, in which kauralexin A3 and abscisic acid were the metabolites most associated with fungal suppression. Therefore, mutants of the *ent-copalyl diphosphate synthase Anther ear 2* (*An2*) gene, which blocked kauralexin biosynthesis, were tested. Those mutants showed increased susceptibility to *F. verticillioides* compared to the wild-type, highlighting the defensive function of acidic terpenoids against *F. verticillioides*. 

Diaz-Gomez [106] used a similar scheme to check the implication of carotenoids in resistance to FER and to contamination with fumonisins. The authors tested the maize inbred M37W, which lacks carotenoids in the endosperm because of the absence of the enzyme phytoene synthase, which is necessary for carotenoid biosynthesis, and a high-carotenoid transformant obtained by introducing maize *phytoene synthase 1* and *Pantoea ananatisphytoene desaturase* genes into M37W. The study reported lower fumonisin levels in the high carotenoid inbred, suggesting that increased carotenoid content reduces fumonisin levels. However, further investigation is needed, because carotenoid-enriched maize lines also differed from their isogenic lines for starch content, and starch composition has been also associated to resistance to fumonisin contamination. In this sense, Blandino and Reyneri had shown in a previous study [107] that an increased content of amylopectin in the starch could stimulate fumonisin contamination.

## 6. Conclusions

Genomic, transcriptomic, and metabolomic studies have confirmed the complexity and polygenic nature of maize resistance to kernel fumonisin contamination. Inheritance of resistance is low due to relevant QTL × environment interactions hindering the efficiency of phenotypic selection. As additive genetic variation for FER and fumonisin accumulation is mostly due to many QTL with minor effects, genomic selection approaches are recommended in breeding programs, although molecular markers significantly associated with resistance could be included as fixed effects in the prediction models of the genotypic value. QTL for maize resistance to FER and fumonisin contamination are scattered across almost all chromosomal bins, and candidate genes have been proposed for some high resolution QTL; genes involved in maize immune response signaling deserving special attention. Although GWAS have contributed to find high-resolution QTL and many candidate genes have been proposed for those QTL, so far, only one gene implicated in resistance to FER has been cloned, the gene *ZmAuxRP1*. 

## Figures and Tables

**Table 1:** Summary of published QTL studies for resistance to Fusarium ear rot (FER) and fumonisin contamination (FUM).

Traits	Type of QTL Mapping	Mapping Population	Number and Type of Markers	Reference
FER	Linkage mapping	238-individuals F_2_	149 RFLP ^5^	Pérez-Brito et al. 2001, [43]
FER	Linkage mapping	206-individuals F_2_	106 RFLP	Pérez-Brito et al. 2001, [43]
FER and FUM	Linkage mapping	213 BC_1_F_1:2_ families from (GE440 × FR1064) × FR1064	105 SSR ^6^	Robertson-Hoyt et al. 2006, [53]
FER and FUM	Linkage mapping	143 RIL ^2^ from NC300 × B104	113 SSR	Robertson-Hoyt et al. 2006, [53]
FER	Linkage mapping	187 RIL from 87-1 × Zong 3	246 SSR	Ding et al. 2008, [54]
FER	Linkage mapping	210 F_2:3_ from BT-1 × Xi502	178 SSR	Chen et al. 2012, [55]
FER	Linkage mapping	250 RIL from BT-1 × N6	207 SSR	Li et al. 2011, [56]
FER	Linkage mapping	201 DH from CML495 × susceptible parent	166 SNP ^7^	Chen et al. 2016, [57]
FER	Linkage mapping	277 F_2:3_ families from CML492 × LPSMT	154 SNP	Chen et al. 2016, [57]
FER	Linkage mapping	268 F_2:3_ families from CML495 × LPSMT t	118 SNP	Chen et al. 2016, [57]
FER	Linkage mapping	272 F_2:3_ families from CML449 × LPSMT	93 SNP	Chen et al. 2016, [57]
FER	GWAS ^1^	854 tropical inbreds	43,424 SNP	Chen et al. 2016, [57]
FER and FUM	Stepwise regression	Four RIL populations from a NAM ^3^	7386 GBS ^8^ markers	Morales et al. 2019, [58]
FER	Linkage mapping	298 RIL from LP4637 × L4674	250 SNP	Giomi et al. 2016, [59]
FER and FUM	Linkage mapping	188 F_2:3_ families from CO441 × CO354	41 SSR and 342 SNP	Maschietto et al. 2017, [60]
FER	Linkage mapping	250 RIL from BT-1 × N6	222 SSR	Wu et al. 2020, [63]
FER	GWAS	265 inbreds	224,152 SSR	Wu et al. 2020, [63]
FER	GWAS	267 inbreds from the “Goodman” association panel	47,445 SNP	Zila et al. 2013, [64]
FER	GWAS	1687 inbreds from the USDA maize gene bank	200,978 SNP	Zila et al. 2014, [65]
FER	GWAS	183 tropical inbreds (85 popcorn inbreds)	267,525 SNP	Coan et al. 2018, [66]
FER	GWAS	242 inbreds	23,153 DArT-seq ^9^ markers	de Jong et al. 2018, [67]
FER	GWAS	339 RIL from a MAGIC ^4^	58,556 SNP	Butrón et al. 2019, [68]
FUM	GWAS	256 inbreds from the “Goodman” association panel	226,446 SNP	Samayoa et al. 2019, [69]

^1^ GWAS: Genome-wide association study; ^2^ RIL: Recombinant inbred line; ^3^ NAM: Nested association mapping population; ^4^ MAGIC: Multi-parent advanced generation intercross; ^5^ RFLP: Restriction fragment length polymorphism; ^6^ SSR: Simple sequence repeat; ^7^ SNP: Single nucleotide polymorphism; ^8^ GBS: Genotyping by sequencing; ^9^ DArt-seq: Diversity Array Technology.

## References

[1] Bhat R.V., Miller J. (1991). Mycotoxins and food supply. Foodnutr. Agric..

[2] Bhatnagar D., Payne G., Klich M., Leslie J.F., Leslie J.F., Logrieco A.F. (2014). Identification of toxigenic *Aspergillus* and *Fusarium* species in the maize grain chain. Mycotoxin Reduction in Grain Chains.

[3] CAST (2003). Mycotoxins: Risks in Plant, Animal, and Human Systems.

[4] Braun M.S., Wink M. (2018). Exposure, occurrence, and chemistry of fumonisins and their cryptic derivatives. Compr. Rev. Food Sci. Food Saf..

[5] Munkvold G.P., Arias S., Taschl I., Gruber-Dorninger C., Serna-Saldivar S.O. (2019). Chapter 9—Mycotoxins in corn: Occurrence, impacts, and management. Corn.

[6] Lanubile A., Maschietto V., Marocco A. (2014). Breeding maize for resistance to mycotoxins. Mycotoxin Reduction in Grain Chains.

[7] Blacutt A.A., Gold S.E., Voss K.A., Gao M., Glenn A.E. (2018). *Fusarium verticillioides*: Advancements in understanding the toxicity, virulence, and niche adaptations of a model mycotoxigenic pathogen of maize. Phytopathology.

[8] Munkvold G.P., Desjardins A.E. (1997). Fumonisins in maizecan—We reduce their occurrence?. Plant Dis..

[9] Marasas W.F. (2001). Discovery and occurrence of the fumonisins: A historical perspective. Environ. Health Perspect..

[10] Voss K.A., Smith G.W., Haschek W.M. (2007). Fumonisins: Toxicokinetics, mechanism of action and toxicity. Anim. Feed Sci. Technol..

[11] Rheeder J.P., Marasas W.F.O., Vismer H.F. (2002). Production of fumonisin analogs by *Fusarium* species. Appl. Environ. Microbiol..

[12] Sun G., Wang S., Hu X., Su J., Huang T., Yu J., Tang L., Gao W., Wang J.-S. (2007). Fumonisin B1 contamination of home-grown corn in high-risk areas for esophageal and liver cancer in China. Food Addit. Contam..

[13] Alizadeh A.M., Rohandel G., Roudbarmohammadi S., Roudbary M., Sohanaki H., Ghiasian S.A., Taherkhani A., Semnani S., Aghasi M. (2012). Fumonisin B1 contamination of cereals and risk of esophageal cancer in a high risk area in northeastern Iran. Asian Pac. J. Cancer Prev..

[14] Gelineau-van Waes J., Starr L., Maddox J., Aleman F., Voss K.A., Wilberding J., Riley R.T. (2005). Maternal fumonisin exposure and risk for neural tube defects: Mechanisms in an in vivo mouse model. Birth Defects Res. Part A Clin. Mol. Teratol..

[15] Missmer S.A., Suarez L., Felkner M., Wang E., Merrill A.H., Rothman K.J., Hendricks K.A. (2006). Exposure to fumonisins and the occurrence of neural tube defects along the Texas-Mexico border. Environ. Health Perspect..

[16] IARC (2002). Fumonisin b_1_. Sometraditional herbalmedicines, somemycotoxins, naphthalene and styrene. 82 Monograph of the International Agency for Research of Cancer on the Evaluation of Carcinogenic Risks to Humans.

[17] FDA Guidance for Industry: Fumonisin Levels in Human Foods and Animal Feeds. https://www.fda.gov/regulatory-information/search-fda-guidance-documents/guidance-industry-fumonisin-levels-human-foods-and-animal-feeds.

[18] Anukul N., Vangnai K., Mahakarnchanakul W. (2013). Significance of regulation limits in mycotoxin contamination in Asia and risk management programs at the national level. J. Food Drug Anal..

[19] 1126/2007/EC (2007). Regulation (EC) no 1126/2007 of 28 September 2007 amending regulation (EC) no 1881/2006 setting maximum levels for certain contaminants in foodstuffs as regards fusarium toxins in maize and maize products. Off. J. Eur. Union.

[20] Pascale M., Visconti A., Chelkowski J. (2002). Ear rot susceptibility and mycotoxin contamination of maize hybrids inoculated with *Fusarium* species under field conditions. Eur. J. Plant Pathol..

[21] Cao A., Santiago R., Ramos A.J., Marin S., Reid L.M., Butron A. (2013). Environmental factors related to fungal infection and fumonisin accumulation during the development and drying of white maize kernels. Int. J. Food Microbiol..

[22] Santiago R., Cao A., Butron A. (2015). Genetic factors involved in fumonisin accumulation in maize kernels and their implications in maize agronomic management and breeding. Toxins.

[23] Afolabi C.G., Ojiambo P.S., Ekpo E.J.A., Menkir A., Bandyopadhyay R. (2007). Evaluation of maize inbred lines for resistance to fusarium ear rot and fumonisin accumulation in grain in tropical Africa. Plant Dis..

[24] Kleinschmidt C.E., Clements M.J., Maragos C.M., Pataky J.K., White D.G. (2005). Evaluation of food-grade dent corn hybrids for severity of fusarium ear rot and fumonisin accumulation in grain. Plant Dis..

[25] Presello D.A., Iglesias J., Botta G., Eyherabide G.H. (2007). Severity of fusarium ear rot and concentration of fumonisin in grain of Argentinian maize hybrids. Crop Prot..

[26] Presello D.A., Reid L.M., Mather D.E. (2004). Resistance of Argentine maize germpiasm to gibberella and fusarium ear rots. Maydica.

[27] Henry W.B., Williams W.P., Windham G.L., Hawkins L.K. (2009). Evaluation of maize inbred lines for resistance to Aspergillus and Fusarium ear rot and mycotoxin accumulation. Agron. J..

[28] Clements M.J., Maragos C.A., Pataky J.K., White D.G. (2004). Sources of resistance to fumonisin accumulation in grain and fusarium ear and kernel rot of corn. Phytopathology.

[29] Clements M.J., White D.G. (2004). Identifying sources of resistance to aflatoxin and fumonisin contamination in corn grain. J. Toxicol. Toxin Rev..

[30] Löffler M., Kessel B., Ouzunova M., Miedaner T. (2011). Covariation between line and testcross performance for reduced mycotoxin concentrations in European maize after silk channel inoculation of two *Fusarium* species. Theor. Appl. Genet..

[31] Löffler M., Kessel B., Ouzunova M., Miedaner T. (2010). Population parameters for resistance to *Fusarium graminearum* and *Fusarium verticillioides* ear rot among large sets of early, mid-late and late maturing European maize (*Zea mays* l.) inbred lines. Theor. Appl. Genet..

[32] Robertson L.A., Kleinschmidt C.E., White D.G., Payne G.A., Maragos C.M., Holland J.B. (2006). Heritabilities and correlations of fusarium ear rot resistance and fumonisin contamination resistance in two maize populations. Crop Sci..

[33] Bolduan C., Miedaner T., Schipprack W., Dhillon B.S., Melchinger A.E. (2009). Genetic variation for resistance to ear rots and mycotoxins contamination in early European maize inbred lines. Crop Sci..

[34] Santiago R., Cao A., Malvar R.A., Reid L.M., Butron A. (2013). Assessment of corn resistance to fumonisin accumulation in a broad collection of inbred lines. Field Crop. Res..

[35] Rose L.J., Mouton M., Beukes I., Flett B.C., van der Vyver C., Viljoen A. (2016). Multi-environment evaluation of maize inbred lines for resistance to fusarium ear rot and fumonisins. Plant Dis..

[36] Balconi C., Berardo N., Locatelli S., Lanzanova C., Torri A., Redaelli R. (2014). Evaluation of ear rot (*Fusarium verticillioides*) resistance and fumonisin accumulation in Italian maize inbred lines. Phytopathol. Mediterr..

[37] Cao A., Butron A., Ramos A.J., Marin S., Souto C., Santiago R. (2014). Assessing white maize resistance to fumonisin contamination. Eur. J. Plant Pathol..

[38] Jouany J.P. (2007). Methods for preventing, decontaminating and minimizing the toxicity of mycotoxins in feeds. Anim. Feed Sci. Technol..

[39] Eller M.S., Holland J.B., Payne G.A. (2008). Breeding for improved resistance to fumonisin contamination in maize. Toxin Rev..

[40] Shephard G.S. (2014). Determination of mycotoxins in maize. Mycotoxin Reduction in Grain Chains.

[41] Löffler M., Miedaner T., Kessel B., Ouzunova M. (2010). Mycotoxin accumulation and corresponding ear rot rating in three maturity groups of European maize inoculated by two fusarium species. Euphytica.

[42] Williams W.P., Windham G.L. (2009). Diallel analysis of fumonisin accumulation in maize. Field Crop. Res..

[43] Pérez-Brito D., Jeffers D., González-de-León D., Khairallah M., Cortés-Cruz M., Velázquez-Cardelas G., Azpíroz-Rivero S., Srinivasan G. (2001). QTL mapping of *Fusarium moniliforme* ear rot resistance in highland maize, Mexico. Agrociencia.

[44] Hung H.-Y., Holland J.B. (2012). Diallel analysis of resistance to fusarium ear rot and fumonisin contamination in maize. Crop Sci..

[45] Butron A., Reid L.M., Santiago R., Cao A., Malvar R.A. (2015). Inheritance of maize resistance to gibberella and fusarium ear rots and kernel contamination with deoxynivalenol and fumonisins. Plant Pathol..

[46] Netshifhefhe N.E.I., Flett B.C., Viljoen A., Rose L.J. (2018). Inheritance and genotype by environment analyses of resistance to *Fusarium verticillioides* and fumonisin contamination in maize F_1_ hybrids. Euphytica.

[47] Eller M.S., Payne G.A., Holland J.B. (2010). Selection for reduced fusarium ear rot and fumonisin content in advanced backcross maize lines and their topcross hybrids. Crop Sci..

[48] Presello D.A., Pereyra A.O., Iglesias J., Fauguel C.M., Sampietro D.A., Eyherabide G.H. (2011). Responses to selection of S_5_ inbreds for broad-based resistance to ear rots and grain mycotoxin contamination caused by *Fusarium spp*. in maize. Euphytica.

[49] Horne D.W., Eller M.S., Holland J.B. (2016). Responses to recurrent index selection for reduced fusarium ear rot and lodging and for increased yield in maize. Crop Sci..

[50] Chiuraise N., Derera J., Yobo K.S., Magorokosho C., Nunkumar A., Qwabe N.F.P. (2016). Progress in stacking aflatoxin and fumonisin contamination resistance genes in maize hybrids. Euphytica.

[51] Gaikpa D.S., Miedaner T. (2019). Genomics-assisted breeding for ear rot resistances and reduced mycotoxin contamination in maize: Methods, advances and prospects. Theor. Appl. Genet..

[52] VanOpdorp N.J., Koehler K.L. (2010). Genetic loci associated with Fusarium ear rot (FKR) resistance in maize and generation of improved FKR resistant maize inbred lines c Agrigenetics. Inc. Patent.

[53] Robertson-Hoyt L.A., Jines M.P., Balint-Kurti P.J., Kleinschmidt C.E., White D.G., Payne G.A., Maragos C.M., Molnar T.L., Holland J.B. (2006). QTL mapping for fusarium ear rot and fumonisin contamination resistance in two maize populations. Crop Sci..

[54] Ding J.-Q., Wang X.-M., Chander S., Yan J.-B., Li J.-S. (2008). QTL mapping of resistance to fusarium ear rot using a RIL population in maize. Mol. Breed..

[55] Chen J.F., Ding J.Q., Li H.M., Li Z.M., Sun X.D., Li J.J., Wang R.X., Dai X.D., Dong H.F., Song W.B. (2012). Detection and verification of quantitative trait loci for resistance to fusarium ear rot in maize. Mol. Breed..

[56] Li Z.M., Ding J.Q., Wang R.X., Chen J.F., Sun X.D., Chen W., Song W.B., Dong H.F., Dai X.D., Xia Z.L. (2011). A new QTL for resistance to fusarium ear rot in maize. J. Appl. Genet..

[57] Chen J., Shrestha R., Ding J.Q., Zheng H.J., Mu C.H., Wu J.Y., Mahuku G. (2016). Genome-wide association study and QTL mapping reveal genomic loci associated with fusarium ear rot resistance in tropical maize germplasm. G3-Genes Genomes Genet..

[58] Morales L., Zila C.T., Mejia D.E.M., Arbelaez M.M., Balint-Kurti P.J., Holland J.B., Nelson R.J. (2019). Diverse components of resistance to *Fusarium verticillioides* infection and fumonisin contamination in four maize recombinant inbred families. Toxins.

[59] Giomi G.M., Kreff E.D., Iglesias J., Fauguel C.M., Fernandez M., Oviedo M.S., Presello D.A. (2016). Quantitative trait loci for fusarium and gibberella ear rot resistance in Argentinian maize germplasm. Euphytica.

[60] Maschietto V., Colombi C., Pirona R., Pea G., Strozzi F., Marocco A., Rossini L., Lanubile A. (2017). QTL mapping and candidate genes for resistance to fusarium ear rot and fumonisin contamination in maize. BMC Plant Biol..

[61] Xiang K., Zhang Z.M., Reid L.M., Zhu X.Y., Yuan G.S., Pan G.T. (2010). A meta-analysis of QTL associated with ear rot resistance in maize. Maydica.

[62] Morales L., Marino T.P., Wenndt A.J., Fouts J.Q., Holland J.B., Nelson R.J. (2018). Dissecting symptomatology and fumonisin contamination produced by *Fusarium verticillioides* in maize ears. Phytopathology.

[63] Wu Y.B., Zhou Z.J., Dong C.P., Chen J.F., Ding J.Q., Zhang X.C., Mu C., Chen Y.N., Li X.P., Li H.M. (2020). Linkage mapping and genome-wide association study reveals conservative QTL and candidate genes for fusarium rot resistance in maize. BMC Genom..

[64] Zila C.T., Fernando Samayoa L., Santiago R., Butron A., Holland J.B. (2013). A genome-wide association study reveals genes associated with fusarium ear rot resistance in a maize core diversity panel. G3-Genes Genomes Genet..

[65] Zila C.T., Ogut F., Romay M.C., Gardner C.A., Buckler E.S., Holland J.B. (2014). Genome-wide association study of fusarium ear rot disease in the U.S.A. maize inbred line collection. BMC Plant Biol..

[66] Coan M.M.D., Senhorinho H.J.C., Pinto R.J.B., Scapim C.A., Tessmann D.J., Williams W.P., Warburton M.L. (2018). Genome-wide association study of resistance to ear rot by *Fusarium verticillioides* in a tropical field maize and popcorn core collection. Crop Sci..

[67] de Jong G., Pamplona A.K.A., Von Pinho R.G., Balestre M. (2018). Genome-wide association analysis of ear rot resistance caused by *Fusarium verticillioides* in maize. Genomics.

[68] Butron A., Santiago R., Cao A., Samayoa L.F., Malvar R.A. (2019). QTLs for resistance to fusarium ear rot in a multiparent advanced generation intercross (MAGIC) maize population. Plant Dis..

[69] Samayoa L.F., Cao A., Santiago R., Malvar R.A., Butron A. (2019). Genome-wide association analysis for fumonisin content in maize kernels. BMC Plant Biol..

[70] Munkvold G.P., McGee D.C., Carlton W.M. (1997). Importance of different pathways for maize kernel infection by *Fusarium moniliforme*. Phytopathology.

[71] Munkvold G.P., Carlton W.M. (1997). Influence of inoculation method on systemic *Fusarium moniliforme* infection of maize plants grown from infected seeds. Plant Dis..

[72] Venturini G., Assante G., Vercesi A. (2011). *Fusarium verticillioides* contamination patterns in northern Italian maize during the growing season. Phytopathol. Mediterr..

[73] Oren L., Ezrati S., Cohen D., Sharon A. (2003). Early events in the *Fusarium verticillioides*-maize interaction characterized by using a green fluorescent protein-expressing transgenic isolate. Appl. Environ. Microbiol..

[74] Ju M., Zhou Z.J., Mu C., Zhang X.C., Gao J.Y., Liang Y.K., Chen J.F., Wu Y.B., Li X.P., Wang S.W. (2017). Dissecting the genetic architecture of *Fusarium verticillioides* seed rot resistance in maize by combining QTL mapping and genome-wide association analysis. Sci. Rep..

[75] Septiani P., Lanubile A., Stagnati L., Busconi M., Nelissen H., Pe M.E., Dell’Acqua M., Marocco A. (2019). Unravelling the genetic basis of fusarium seedling rot resistance in the magic maize population: Novel targets for breeding. Sci. Rep..

[76] Stagnati L., Lanubile A., Samayoa L.F., Bragalanti M., Giorni P., Busconi M., Holland J.B., Marocco A. (2019). A genome wide association study reveals markers and genes associated with resistance to *Fusarium verticillioides* infection of seedlings in a maize diversity panel. G3 (BethesdaMd.).

[77] Mu C., Gao J.Y., Zhou Z.J., Wang Z., Sun X.D., Zhang X.C., Dong H.F., Han Y.A., Li X.P., Wu Y.B. (2019). Genetic analysis of cob resistance to *F. verticillioides*: Another step towards the protection of maize from ear rot. Theor. Appl. Genet..

[78] Ye J.R., Zhong T., Zhang D.F., Ma C.Y., Wang L.N., Yao L.S., Zhang Q.Q., Zhu M., Xu M.L. (2019). The auxin-regulated protein ZmAuxRP1 coordinates the balance between root growth and stalk rot disease resistance in maize. Mol. Plant..

[79] Glenn A.E., Hinton D.M., Yates I.E., Bacon C.W. (2001). Detoxification of corn antimicrobial compounds as the basis for isolating *Fusarium verticillioides* and some other *Fusarium* species from corn. Appl. Environ. Microbiol..

[80] Lanubile A., Maschietto V., Borrelli V.M., Stagnati L., Logrieco A.F., Marocco A. (2017). Molecular basis of resistance to fusarium ear rot in maize. Front. Plant Sci..

[81] Lanubile A., Pasini L., Marocco A. (2010). Differential gene expression in kernels and silks of maize lines with contrasting levels of ear rot resistance after *Fusarium verticillioides* infection. J. Plant Physiol..

[82] Lanubile A., Bernardi J., Marocco A., Logrieco A., Paciolla C. (2012). Differential activation of defense genes and enzymes in maize genotypes with contrasting levels of resistance to *Fusarium verticillioides*. Environ. Exp. Bot..

[83] Lanubile A., Bernardi J., Battilani P., Logrieco A., Marocco A. (2012). Resistant and susceptible maize genotypes activate different transcriptional responses against *Fusarium verticillioides*. Physiol. Mol. Plant Pathol..

[84] Yuan G.S., Zhang Z.M., Xiang K., Zhao M.J., Shen Y.O., Pan G.T. (2012). Large-scale identification of differentially expressed genes in maize inbreds susceptible and resistant to fusarium ear rot. Plant Omics.

[85] Yuan G.S., Zhang Z.M., Xiang K., Shen Y.O., Du J., Lin H.J., Liu L., Zhao M.J., Pan G.T. (2013). Different gene expressions of resistant and susceptible maize inbreds in response to *Fusarium verticillioides* infection. Plant Mol. Biol. Rep..

[86] Campos-Bermudez V.A., Fauguel C.M., Tronconi M.A., Casati P., Presello D.A., Andreo C.S. (2013). Transcriptional and metabolic changes associated to the infection by *Fusarium verticillioides* in maize inbreds with contrasting ear rot resistance. PLoS ONE.

[87] Lanubile A., Ferrarini A., Maschietto V., Delledonne M., Marocco A., Bellin D. (2014). Functional genomic analysis of constitutive and inducible defense responses to *Fusarium verticillioides* infection in maize genotypes with contrasting ear rot resistance. BMC Genom..

[88] Wang Y.P., Zhou Z.J., Gao J.Y., Wu Y.B., Xia Z.L., Zhang H.Y., Wu J.Y. (2016). The mechanisms of maize resistance to *Fusarium verticillioides* by comprehensive analysis of RNA-seq data. Front. Plant Sci..

[89] Yuan G.S., Xiang K., Zhang Z.M., Shen Y.O., Du J., Lin H.J., Liu L., Pan G.T. (2013). Analysis on the relationship between *Fusarium verticillioides* infection-induced genes and ear rot resistance in maize. J. Food Agric. Environ..

[90] Bravo J.M., Campo S., Murillo I., Coca M., Segundo B.S. (2003). Fungus- and wound-induced accumulation of mRNA containing a class ii chitinase of the pathogenesis-related protein 4 (PR-4) family of maize. Plant Mol. Biol..

[91] Murillo I., Cavallarin L., San Segundo B. (1999). Cytology of infection of maize seedlings by *Fusarium moniliforme* and immunolocalization of the pathogenesis-related PRMs protein. Phytopathology.

[92] Maschietto V., Lanubile A., De Leonardis S., Marocco A., Paciolla C. (2016). Constitutive expression of pathogenesis-related proteins and antioxydant enzyme activities triggers maize resistance towards *Fusarium verticillioides*. J. Plant Physiol..

[93] Lanubile A., Maschietto V., De Leonardis S., Battilani P., Paciolla C., Marocco A. (2015). Defense responses to mycotoxin-producing fungi *Fusarium proliferatum*, *F. subglutinans*, and *Aspergillus flavus* in kernels of susceptible and resistant maize genotypes. Mol. Plant-Microbe Interact..

[94] Battilani P., Lanubile A., Scala V., Reverberi M., Gregori R., Falavigna C., Dall’asta C., Park Y.S., Bennett J., Borrego E.J. (2018). Oxylipins from both pathogen and host antagonize jasmonic acid-mediated defence via the 9-lipoxygenase pathway in *Fusarium verticillioides* infection of maize. Mol. Plant Pathol..

[95] Maschietto V., Marocco A., Malachova A., Lanubile A. (2015). Resistance to *Fusarium verticillioides* and fumonisin accumulation in maize inbred lines involves an earlier and enhanced expression of *lipoxygenase* (*lox*) genes. J. Plant Physiol..

[96] Lanubile A., Logrieco A., Battilani P., Proctor R.H., Marocco A. (2013). Transcriptional changes in developing maize kernels in response to fumonisin-producing and nonproducing strains of *Fusarium verticillioides*. Plant Sci..

[97] Fauguel C.M., Bermudez V.A.C., Iglesias J., Fernandez M., Farroni A., Andreo C.S., Presello D.A. (2017). Volatile compounds released by maize grains and silks in response to infection by *Fusarium verticillioides* and its association with pathogen resistance. Plant Pathol..

[98] Gao X.Q., Shim W.B., Gobel C., Kunze S., Feussner I., Meeley R., Balint-Kurti P., Kolomiets M. (2007). Disruption of a maize *9-lipoxygenase* results in increased resistance to fungal pathogens and reduced levels of contamination with mycotoxin fumonisin. Mol. Plant-Microbe Interact..

[99] Christensen S.A., Nemchenko A., Park Y.S., Borrego E., Huang P.C., Schmelz E.A., Kunze S., Feussner I., Yalpani N., Meeley R. (2014). The novel monocot-specific *9-lipoxygenase zmlox12* is required to mount an effective jasmonate-mediated defense against *Fusarium verticillioides* in maize. Mol. Plant-Microbe Interact..

[100] Scala V., Beccaccioli M., Dall’Asta C., Giorni P., Fanelli C. (2015). Analysis of the expression of genes related to oxylipin biosynthesis in *Fusarium verticillioides* and maize kernels during their interaction. J. Plant Pathol..

[101] Constantino N.N., Mastouri F., Damarwinasis R., Borrego E.J., Moran-Diez M.E., Kenerley C.M., Gao X.Q., Kolomiets M.V. (2013). Root-expressed maize *lipoxygenase 3* negatively regulates induced systemic resistance to *Colletotrichum graminicola* in shoots. Front. Plant Sci..

[102] Agostini R.B., Postigo A., Rius S.P., Rech G.E., Campos-Bermudez V.A., Vargas W.A. (2019). Long-lasting primed state in maize plants: Salicylic acid and steroid signaling pathways as key players in the early activation of immune responses in silks. Mol. Plant-Microbe Interact..

[103] Atanasova-Penichon V., Barreau C., Richard-Forget F. (2016). Antioxidant secondary metabolites in cereals: Potential involvement in resistance to *Fusarium* and mycotoxin accumulation. Front. Microbiol..

[104] Venturini G., Babazadeh L., Casati P., Pilu R., Salomoni D., Toffolatti S.L. (2016). Assessing pigmented pericarp of maize kernels as possible source of resistance to fusarium ear rot, *Fusarium spp.* infection and fumonisin accumulation. Int. J. Food Microbiol..

[105] Christensen S.A., Sims J., Vaughan M.M., Hunter C., Block A., Willett D., Alborn H.T., Huffaker A., Schmelz E.A. (2018). Commercial hybrids and mutant genotypes reveal complex protective roles for inducible terpenoid defenses in maize. J. Exp. Bot..

[106] Diaz-Gomez J., Marin S., Nogareda C., Sanchis V., Ramos A.J. (2016). The effect of enhanced carotenoid content of transgenic maize grain on fungal colonization and mycotoxin content. Mycotoxin Res..

[107] Blandino M., Reyneri A. (2007). Comparison between normal and waxy maize hybrids for *Fusarium*-toxin contamination in NW Italy. Maydica.

